# Multivariate hematological and biochemical profiling reveals conserved physiological modules in Brahman- and Charolais-crossbred beef cattle under tropical smallholder systems

**DOI:** 10.14202/vetworld.2026.2418-2433

**Published:** 2026-06-13

**Authors:** Somchart Tana, Jirasak Thongseela, Pralongyut Sripalwit, Promporn Raksaseri, Kanta Sangwijit, Paiboon Panase, Payungsuk Intawicha, Sureeporn Saengwong

**Affiliations:** 1 Division of Animal Science, School of Agriculture and Natural Resources, University of Phayao, Phayao, 56000, Thailand; 2 Department of Biology, School of Science, University of Phayao, Phayao, 56000, Thailand; 3 Department of Anatomy, Faculty of Veterinary Science, Chulalongkorn University, Bangkok 10330, Thailand; 4 Plasma Bioengineering Unit, School of Science, University of Phayao, Phayao, 56000, Thailand; 5 Division of Fisheries, School of Agriculture and Natural Resources, University of Phayao, Phayao, 56000, Thailand

**Keywords:** biochemical profiling, Brahman-crossbred cattle, Charolais-crossbred cattle, hematological profiling, multivariate analysis, physiological modules, smallholder systems, tropical beef cattle

## Abstract

**Background and Aim:**

Hematological and biochemical profiling is widely used to evaluate the physiological and health status of cattle; however, most studies interpret blood parameters individually and may overlook coordinated physiological regulation. Multivariate analytical approaches can reveal biologically integrated relationships among blood traits and provide a broader understanding of systemic physiology. Therefore, this study aimed to identify coordinated physiological modules, describe multivariate relationships among hematological and biochemical traits, and quantify breed-related physiological variation in Brahman- and Charolais-crossbred beef cattle reared under tropical smallholder production systems in northern Thailand.

**Materials and Methods:**

A cross-sectional study was conducted using 97 clinically healthy breeding beef cows comprising 74 Brahman-crossbred and 23 Charolais-crossbred cattle aged 3–6 years. Blood samples were collected from smallholder farms in Phayao, Chiang Rai, and Lampang provinces, Thailand. Hematological and biochemical parameters were analyzed using automated veterinary analyzers. Univariate analyzes were performed to compare breed-specific differences, while multivariate analyzes, including hierarchical clustering, principal component analysis (PCA), correlation heatmaps, and permutational multivariate analysis of variance (PERMANOVA), were applied to determine physiological relationships and breed contributions to overall blood profile variation.

**Results:**

Hierarchical clustering identified six physiologically coherent modules consisting of erythrocyte, platelet, leukocyte, protein metabolism, hepatic enzyme, and renal function traits, demonstrating coordinated systemic regulation rather than isolated parameter variation. PCA showed that the first two principal components explained 31.1% of the total variance and represented erythrocyte–renal and immune–hepatic physiological axes. Substantial overlap in multivariate physiological space was observed between Brahman- and Charolais-crossbred cattle. PERMANOVA indicated a statistically significant but small breed effect (R² = 0.021, p = 0.028), suggesting that environmental and management factors exerted greater influence on physiological organization than breed background. Breed-related differences were primarily associated with monocyte indices, erythrocyte distribution indices, albumin, and hepatic enzyme activity.

**Conclusion:**

This study demonstrated that hematological and biochemical traits in tropical crossbred beef cattle are organized into conserved physiological modules reflecting integrated systemic regulation. The findings support the application of modular blood profiling as a practical and biologically meaningful approach for herd health monitoring and resilience assessment in tropical smallholder production systems.

## INTRODUCTION

Production of beef cattle contributes substantially to food security and supports rural agricultural sustainability in tropical regions. Tropical smallholder production systems commonly rear beef cattle under heterogeneous management conditions, with marked variations in feed availability, environmental pressure, and veterinary care [1–3]. Under such conditions, fluctuations in animal health status and productive performance frequently occur, making herd health management challenging. Therefore, reliable, practical, and biologically meaningful indicators are required to improve health monitoring and management efficiency in tropical smallholder beef production systems [4–6]. One of the most informative indicators is the blood profile, which comprises hematological and biochemical parameters that can be used to evaluate physiological status, nutritional condition, metabolic balance, and disease prevalence in livestock [7]. Baseline blood profiles representing normal physiological conditions can be established and subsequently used as reference values to detect health disturbances [8, 9]. These measurements may assist in disease diagnosis, prognosis evaluation, and monitoring treatment effectiveness [10]. Moreover, blood metabolites provide important information regarding energy, protein, and mineral metabolism and reflect the nutritional and physiological responses of animals to their environment [11, 12].

In veterinary medicine, hematological and biochemical parameters are routinely used to obtain an objective assessment of physiological status, metabolic homeostasis, and organ function [13]. Routine blood profiling enables evaluation of nutritional sufficiency, stress responses, health condition, and early subclinical disorders before overt clinical manifestations appear, thereby contributing to improved productive performance and herd management [14]. Furthermore, blood-based assessments can be applied to monitor adaptation to environmental stressors and evaluate management practices under different production systems [4, 15]. However, blood parameters should not be interpreted individually because physiological processes such as erythropoiesis, immune regulation, hepatic metabolism, renal clearance, and protein homeostasis are interconnected and often change simultaneously. Consequently, interpretation based solely on univariate analysis may oversimplify complex biological interactions and fail to capture coordinated physiological regulation. This limitation is particularly important in beef cattle raised under tropical environments, where animals are continuously exposed to heat stress, nutritional variation, and parasitic challenges [16, 17]. Previous studies have primarily focused on comparing mean values of individual blood parameters among breeds, age groups, or management systems [7, 18]. Although such approaches provide valuable descriptive information, they do not adequately explain how physiological variables interact within broader systemic regulatory networks. As a result, relationships among hematological and biochemical pathways, compensatory mechanisms, and integrated physiological responses to environmental or genetic factors may remain unclear. This issue is especially relevant in crossbred cattle populations because physiological phenotypes may reflect both conserved regulatory mechanisms and breed-specific adaptive responses [19, 20].

Despite the increasing application of blood-based biomarkers in cattle health assessment, important research gaps remain regarding the multivariate physiological organization of hematological and biochemical traits in tropical beef cattle. Most previous multivariate studies have been conducted in dairy cattle reared under temperate production systems or have focused primarily on productivity-associated traits rather than physiological resilience and systemic regulation [21–23]. Furthermore, limited information is available regarding how blood parameters are integrated into coordinated physiological modules under heterogeneous tropical smallholder environments, where environmental stressors such as heat, fluctuating nutrition, and parasitic exposure are expected to induce complex systemic responses rather than isolated alterations [19]. To date, no study has systematically characterized the higher-order physiological structure of blood profiles in tropical crossbred beef cattle using integrated multivariate analytical approaches. Therefore, understanding the coordinated relationships among hematological and biochemical parameters is essential for improving interpretation of blood profiles and developing biologically meaningful herd health monitoring strategies in tropical production systems.

Multivariate analytical approaches provide an effective framework for simultaneously evaluating multiple physiological parameters and identifying biologically interconnected patterns [21–24]. Methods such as correlation analysis, principal component analysis (PCA), and hierarchical clustering can identify associations among variables, characterize physiological variance, and classify groups of related traits into functional biological modules [24]. In veterinary physiology, these approaches facilitate systemic interpretation of blood profiles and improve understanding of animal health, resilience, productivity, and disease susceptibility at the herd-level [14, 25]. Such approaches are particularly valuable in tropical beef cattle systems, where multiple environmental and physiological factors interact simultaneously.

Brahman and Charolais cattle are commonly used in tropical crossbreeding programs because each breed possesses desirable adaptive and productive characteristics [26, 27]. Brahman cattle demonstrate superior heat tolerance and environmental adaptability, whereas Charolais cattle are recognized for their favorable growth performance and carcass traits. Crossbred cattle derived from these breeds are widely used in tropical production systems because of their enhanced adaptability and productive potential. Nevertheless, crossbreeding may also generate unique physiological phenotypes that differ substantially from those of the parental breeds. Consequently, establishing physiological patterns and multivariate relationships among hematological and biochemical parameters in crossbred cattle is necessary for accurate interpretation of blood profiles and effective herd health surveillance under tropical smallholder conditions [21, 22]. However, empirical evidence regarding these integrated physiological relationships in tropical crossbred beef cattle remains limited [28].

Aim of the study

Therefore, the present study was conducted to address these knowledge gaps. Specifically, this study aimed to: (1) identify groups of correlated hematological and biochemical parameters using multivariate analyzes; (2) characterize the multivariate relationships among hematological and biochemical parameters in crossbred beef cattle reared under tropical smallholder systems; and (3) quantify the contribution of breed to multivariate physiological variation and localized biomarker differences, thereby establishing a novel modular framework for interpretation of blood profiles in tropical beef production systems.

## MATERIALS AND METHODS

### Ethical approval

All procedures involving animals were reviewed and approved by the Institutional Animal Care and Use Committee, University of Phayao (IACUC UP), Thailand, under approval number 1-017-67. The study was conducted in accordance with institutional guidelines for the ethical use of animals in research and followed the principles of animal welfare, including minimizing discomfort, stress, and unnecessary handling.

Before sampling, all cattle were clinically examined by a licensed veterinarian, and only apparently healthy animals were included. Blood collection was performed by licensed veterinarians using standard aseptic techniques. Animals were gently restrained for the shortest possible duration, and blood samples were collected from the jugular vein within approximately 2 min of handling to minimize stress. No experimental treatment, invasive intervention beyond routine venipuncture, or procedure causing pain or long-term harm was performed. The study followed the ARRIVE 2.0 guidelines where applicable.

### Study period and location

**Figure 1:**
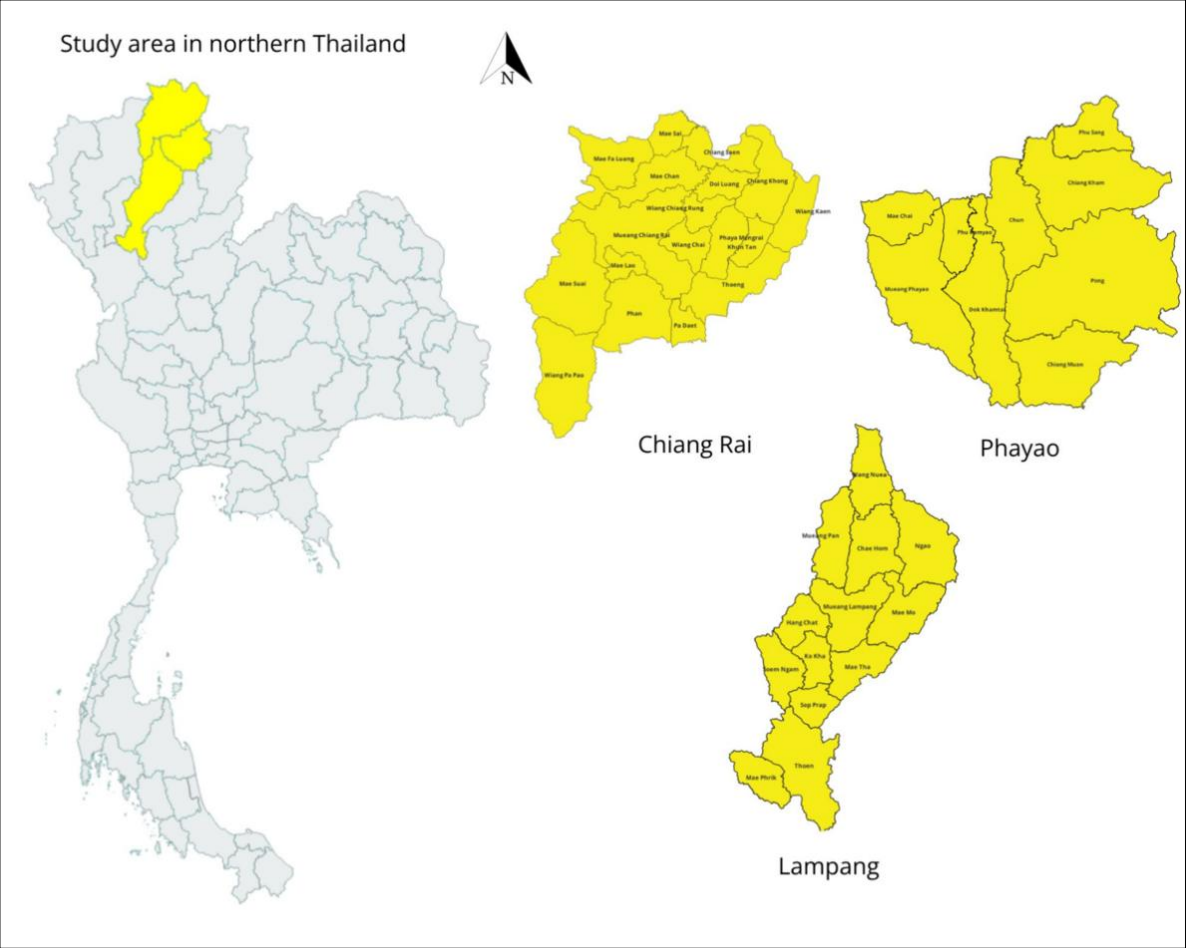
Study area showing the locations of the three provinces included in the study (Chiang Rai, Phayao, and Lampang) in northern Thailand. The map was generated using QGIS 4.0.

The study was conducted between October and November 2024 on smallholder cattle farms located in Phayao, Chiang Rai, and Lampang provinces in northern Thailand (Figure 1). The study area is characterized by a tropical savanna climate (Köppen Aw) with distinct wet (May–October) and dry (November–April) seasons. The annual average ambient temperature ranges from 25°C to 28°C and may increase to approximately 35°C during the hot season. The participating farms represented typical tropical smallholder beef production systems in northern Thailand, where herds generally consisted of 5–15 cattle managed under semi-extensive conditions. Animals were commonly grazed on natural grasslands and supplemented with crop residues, including rice straw, together with limited amounts of commercial concentrate feed.

### Study design

This study employed a cross-sectional observational design to evaluate hematological and biochemical profiles in crossbred beef cattle reared under tropical smallholder production systems. The study focused on identifying multivariate physiological relationships and breed-associated variation among blood parameters under field conditions. Animals from multiple smallholder farms were included to capture physiological variability associated with heterogeneous environmental and management conditions commonly encountered in tropical production systems.

### Animals

A total of 97 apparently healthy breeding beef cows comprising 74 Brahman-crossbred cattle (*Bos indicus* influence) and 23 Charolais-crossbred cattle (*Bos taurus* influence) aged 3–6 years were included in the study. Mean age did not differ significantly between groups (Brahman-crossbred: 3.89 ± 0.80 years; Charolais-crossbred: 4.13 ± 1.01 years; Wilcoxon test, p = 0.4008). Animals were selected from smallholder farms and clinically examined by a licensed veterinarian before sampling. Only animals without apparent clinical signs of disease were included in the study. None of the animals had received vaccination or anthelmintic treatment within 30 days before blood collection. All cattle were managed under typical northern Thai smallholder conditions involving daytime grazing on natural pastures supplemented with crop residues, particularly rice straw, and occasional commercial concentrate supplementation.

### Blood sampling and laboratory analyses

Blood samples were collected between October and November 2024. Approximately 10 mL of blood was collected from the jugular vein using an 18–20 G needle into vacuum collection tubes consisting of one 5 mL K3- Ethylenediaminetetraacetic acid (EDTA) tube for hematological analysis and one 5 mL serum-separation tube for biochemical analysis. Sampling was performed between 07:00 and 11:00 h to minimize diurnal variation, while animals were minimally restrained during handling. Tubes were gently inverted 8–10 times immediately after collection.

EDTA blood samples were stored at 4°C and analyzed within 4 h after collection. Serum tubes were allowed to clot at room temperature (25°C) for 30–60 min and subsequently centrifuged at 3,000 rpm (approximately 1,500 × *g*) for 15 min. Serum samples were visually inspected for hemolysis, lipemia, and icterus before analysis. Samples not immediately processed were stored at −20°C for a maximum of 48 h. Hemolyzed or lipemic samples were excluded or appropriately documented.

Hematological parameters including red blood cell (RBC), hemoglobin (HGB), hematocrit (HCT), mean corpuscular volume (MCV), mean corpuscular HGB (MCH), mean corpuscular HGB concentration (MCHC), red cell distribution width–coefficient of variation (RDW-CV), red cell distribution width–standard deviation (RDW-SD), white blood cell (WBC), differential leukocyte counts, platelet (PLT), mean platelet volume (MPV), plateletcrit (PCT), and platelet distribution width (PDW) were measured using an automated veterinary hematology analyzer (BC-5300 Vet; Mindray Bio-Medical Electronics Co., Shenzhen, China) according to the manufacturer’s instructions.

Biochemical parameters including alanine aminotransferase (ALT), aspartate aminotransferase (AST), alkaline phosphatase (ALP), blood urea nitrogen (BUN), creatinine (CREA), total protein (TP), albumin (ALB), and globulin (GLOB) were analyzed using an automated chemistry analyzer (BioMajesty JCA-BM6010/C; JEOL Ltd., Tokyo, Japan) together with commercial reagent kits (DiaSys Diagnostic Systems GmbH, Holzheim, Germany) according to the manufacturer’s instructions. Quality control procedures were performed daily using two-level control sera, and intra- and inter-assay coefficients of variation were maintained below 5% for all analytes. All laboratory analyses were conducted at the Veterinary Diagnostic Laboratory, Small Animal Teaching Hospital, Faculty of Veterinary Medicine, Chiang Mai University, Thailand.

### Statistical analysis

A total of 31 hematological and biochemical variables were included in the final dataset and analyzed using both univariate and multivariate statistical approaches. Hematological and biochemical parameters of Brahman- and Charolais-crossbred cattle were compared according to breed. Data completeness was verified before analysis, and descriptive statistics were calculated for each breed group. No missing values were detected in the dataset.

Normality of the data distribution was assessed using the Shapiro-Wilk test, whereas homogeneity of variance was evaluated using Levene’s test. Independent-samples t-tests were used to compare normally distributed variables between breeds, whereas Mann-Whitney U tests were applied to non-normally distributed variables. Multiple comparison adjustment was not performed because breed comparisons were considered exploratory. However, effect sizes (Hedges’ g) together with 95% confidence intervals were calculated to facilitate interpretation of breed-associated differences.

Pearson correlation matrixes were visualized as clustered heatmaps to evaluate relationships among variables. Multivariate analyses were performed using standardized z-score values for all variables. Hierarchical clustering based on the complete linkage method and correlation matrixes was conducted to identify physiological modules among hematological and biochemical parameters.

PCA was performed using correlation matrixes to investigate multivariate physiological trends among hematological and biochemical parameters. Components with eigenvalues >1 together with biologically meaningful variance identified through scree plot evaluation were retained for interpretation.

Permutational multivariate analysis of variance (PERMANOVA) implemented in the vegan R package was performed using Euclidean distance matrixes of standardized variables to evaluate multivariate breed-associated differences. A total of 9,999 permutations were conducted. Data quality was additionally evaluated using graphical methods including boxplots and PCA score plots. No extreme outliers were identified; therefore, no observations were excluded from the analyses.

The unequal sample sizes reflected the lower availability of Charolais-crossbred cattle in smallholder production systems within the study area. Considering the relatively small effect-size identified in the PERMANOVA analysis, the study may have had limited statistical power to detect subtle breed-associated physiological differences. Nevertheless, the integrated analytical workflow combining hierarchical clustering, PCA, and PERMANOVA provided a systems-physiology framework rarely applied to field-based beef cattle datasets obtained under tropical production conditions.

All statistical analyses were performed using R statistical software version 4.5.1 (R Core Team, 2025). PCA was conducted using the FactoMineR package, correlation heatmaps were generated using the pheatmap and corrplot packages, and PERMANOVA was performed using the vegan package. Statistical significance was established at p < 0.05.

## RESULTS

### Hematological and biochemical profiles of Brahman- and Charolais-crossbred cattle

The hematological profiles of Brahman- and Charolais-crossbred cattle are presented in Table 1. Among the evaluated hematological variables, only monocyte-associated indices differed significantly between breeds. Mon% was significantly higher in Charolais-crossbred cattle (7.89% ± 4.71%) than in Brahman-crossbred cattle (4.76% ± 3.55%; p = 0.005). Similarly, Mon# was significantly higher in Charolais-crossbred cattle (1.04 ± 0.78 × 10³/µL) compared with Brahman-crossbred cattle (0.56 ± 0.50 × 10³/µL; p = 0.003). No significant differences were observed for erythrocyte, platelet, or other leukocyte-related parameters between breeds.

**Table 1:** Descriptive statistics of hematological parameters in Brahman- and Charolais-crossbred cattle.

Parameter	Brahman-crossbred cattle (n = 74)				Charolais-crossbred cattle (n = 23)				p-value

	Mean ± SD	Median	Min	Max	Mean ± SD	Median	Min	Max	
RBC (× 10⁶/µL)	6.78 ± 1.31	6.80	2.65	9.95	6.42 ± 1.19	6.49	3.08	8.09	0.227
HGB (g/dL)	10.57 ± 2.25	10.20	5.40	18.70	9.89 ± 1.72	9.90	5.10	12.50	0.362
HCT (%)	32.1 ± 6.6	30.8	17.0	53.7	30.4 ± 4.9	30.3	16.1	38.2	0.466
MCV (fL)	47.6 ± 5.8	46.3	37.1	64.3	47.7 ± 3.8	47.3	38.9	54.0	0.503
MCH (pg)	15.7 ± 1.9	15.4	11.5	20.8	15.5 ± 1.3	15.7	11.7	17.4	0.690
MCHC (g/dL)	33.0 ± 1.2	33.2	29.5	34.9	32.5 ± 1.1	32.6	30.1	34.4	0.078
WBC (10³ cells/mL)	11.65 ± 6.17	10.59	4.63	45.83	12.83 ± 7.28	10.52	4.92	38.79	0.500
Neu (%)	20.9 ± 15.7	19.8	0.3	58.0	18.6 ± 10.1	18.7	0.5	45.7	0.423
Lym (%)	71.13 ± 19.25	69.75	32.80	99.40	71.22 ± 13.63	68.90	46.30	99.30	0.959
Mon (%)	4.76 ± 3.55	4.70	0.00	14.10	7.89 ± 4.71	7.00	0.20	17.70	0.005
Eos (%)	3.03 ± 3.94	1.95	0.00	22.00	2.10 ± 2.74	1.10	0.00	12.00	0.316
Bas (%)	0.18 ± 0.67	0.00	0.00	4.70	0.15 ± 0.21	0.10	0.00	0.90	0.066
RDW-CV (%)	18.0 ± 1.7	17.9	14.5	22.5	19.5 ± 4.3	18.6	16.1	37.5	0.119
RDW-SD (fL)	35.8 ± 5.9	34.6	25.8	54.8	38.9 ± 9.1	36.8	30.1	74.7	0.069
PLT (10³ cells/mL)	234.6 ± 203.5	181.5	19.0	1363.0	238.6 ± 243.4	149.0	23.0	704.0	0.281
MPV (%)	6.28 ± 0.50	6.30	5.10	7.30	6.11 ± 0.74	6.20	4.90	7.80	0.314
PDW (%)	15.17 ± 0.53	15.15	13.90	17.10	14.98 ± 0.42	15.00	14.00	15.70	0.116
PCT (%)	0.14 ± 0.12	0.11	0.01	0.79	0.13 ± 0.13	0.09	0.01	0.38	0.254
Neu# (× 10³/µL)	2.66 ± 2.88	2.29	0.02	17.42	2.58 ± 2.23	2.21	0.05	10.24	0.738
Lym# (× 10³/µL)	8.00 ± 3.77	7.50	2.15	23.38	8.82 ± 4.33	7.71	4.20	23.47	0.435
Mon# (× 10³/µL)	0.56 ± 0.50	0.50	0.00	2.84	1.04 ± 0.78	0.99	0.01	3.28	0.003
Eos# (× 10³/µL)	0.03 ± 0.70	0.18	0.00	3.66	0.36 ± 0.68	0.12	0.00	2.95	0.422
Bas# (× 10³/µL)	0.03 ± 0.16	0.00	0.00	1.22	0.28 ± 0.05	0.01	0.00	0.18	0.063

The data are presented as mean ± standard deviation (SD), median, minimum (Min), and maximum (Max). Group comparisons were performed using independent-samples t-tests for normally distributed variables or Mann-Whitney U tests for non-normally distributed variables. Bas = basophils, Eos = eosinophils, HCT = hematocrit, HGB = hemoglobin, Lym = lymphocytes, MCH = mean corpuscular HGB, MCHC = mean corpuscular HGB concentration, MCV = mean corpuscular volume, Mon = monocytes, MPV = mean platelet volume, Neu = neutrophils, PCT = plateletcrit, PDW = platelet distribution width, PLT = platelet count, RBC = red blood cell, RDW-CV = red cell distribution width–coefficient of variation, RDW-SD = red cell distribution width–standard deviation, WBC = white blood cell.

Biochemical parameters of Brahman- and Charolais-crossbred cattle are summarized in Table 2. Similar to hematological findings, only a limited number of biochemical variables differed significantly between breeds. ALB concentration was significantly higher in Brahman-crossbred cattle (2.99 ± 0.42 g/dL) than in Charolais-crossbred cattle (2.74 ± 0.41 g/dL; p = 0.015). In contrast, AST activity was significantly higher in Charolais-crossbred cattle (79.4 ± 49.7 U/L) compared with Brahman-crossbred cattle (63.4 ± 29.8 U/L; p = 0.017). No significant breed-associated differences were observed for ALT, BUN, CREA, GLOB, ALP, or TP.

**Table 2:** Descriptive statistics of biochemical parameters in Brahman- and Charolais-crossbred cattle.

Parameter	Brahman-crossbred cattle (n = 74)				Charolais-crossbred cattle (n = 23)				p-value

	Mean ± SD	Median	Min	Max	Mean ± SD	Median	Min	Max	
ALT (U/L)	11.3 ± 3.7	11.60	2.70	22.10	9.9 ± 4.1	10.00	2.40	17.8	0.112
BUN (mg/dL)	1.69 ± 0.33	1.64	0.91	2.83	1.64 ± 0.26	1.59	1.18	2.26	0.623
CREA (mg/dL)	20.0 ± 7.5	19.0	8.0	39.0	19.0 ± 6.5	17.0	11.0	34.0	0.516
ALB (g/dL)	2.99 ± 0.42	3.00	2.00	4.20	2.74 ± 0.41	2.90	1.90	3.50	0.015
GLOB (g/dL)	5.35 ± 1.17	5.25	2.90	9.10	5.82 ± 1.26	6.00	4.00	8.60	0.100
AST (U/L)	63.4 ± 29.8	57.5	27.0	213.0	79.4 ± 49.7	66.0	32.0	285.0	0.017
ALP (U/L)	120.5 ± 47.6	114.5	37.0	257.0	103.0 ± 46.2	93.0	46.0	233.0	0.068
TP (g/dL)	8.35 ± 1.13	8.30	5.90	11.40	8.56 ± 1.23	8.60	6.50	10.60	0.437

The data are presented as mean ± standard deviation (SD), median, minimum (Min), and maximum (Max). Group comparisons were performed using independent-samples t-tests for normally distributed variables or Mann-Whitney U tests for non-normally distributed variables. ALB = albumin, ALP = alkaline phosphatase, ALT = alanine aminotransferase, AST = aspartate aminotransferase, BUN = blood urea nitrogen, CREA = creatinine, GLOB = globulin, TP = total protein.

Given the limited breed-associated differences observed in the univariate analyses, subsequent analyses focused on evaluating multivariate relationships among hematological and biochemical variables.

### Multivariate correlation analysis and physiological modules

**Figure 2:**
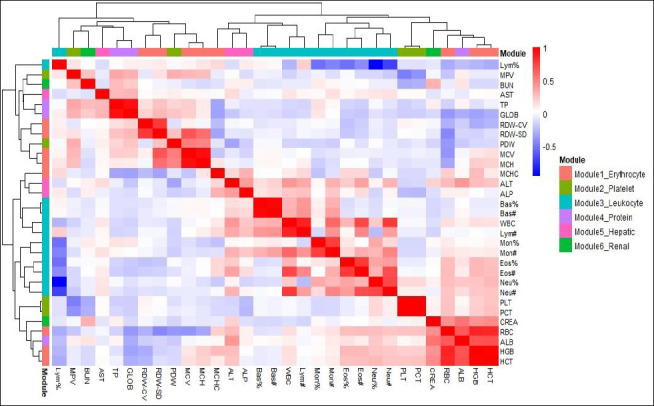
Clustered Pearson correlation heatmap illustrating multivariate relationships among 31 hematological and biochemical variables in crossbred beef cattle. Pearson correlation coefficients were clustered using the Ward hierarchical clustering method. Colors ranged from dark red (strong positive correlation) to dark blue (strong negative correlation), indicating correlation strength and direction. Dendrograms illustrate trait–trait clustering topology. Six physiological modules (erythrocyte, platelet, leukocyte, protein metabolism, hepatic enzyme, and renal) are highlighted using different colors.

The clustered Pearson correlation heatmap revealed well-structured multivariate relationships among the 31 hematological and biochemical parameters (Figure 2). Hierarchical clustering of the trait–trait correlation matrix identified four major clusters with multiple sub-branches, resulting in six physiologically relevant modules. These findings demonstrated that blood profile variation was organized into coordinated physiological networks rather than random independent variables.

Three modules were associated primarily with hematological regulation. The erythrocyte module (Module 1: RBC, HGB, HCT, MCV, MCH, MCHC, RDW-CV, and RDW-SD) represented coordinated erythropoiesis, HGB concentration, and oxygen-carrying capacity. The platelet module (Module 2: PLT, PCT, PDW, and MPV) reflected hemostatic regulation and platelet-associated activity. The leukocyte module (Module 3: WBC count, Lym%, Lym#, Neu%, Neu#, Mon%, Mon#, Eos%, Eos#, Bas%, and Bas#) represented coordinated innate and adaptive immune functions together with their hematopoietic origins.

Biochemical parameters were similarly organized into three physiological modules. Module 4 (TP, ALB, and GLOB) corresponded to protein metabolism and nutritional status. Module 5 (AST, ALT, and ALP) represented hepatic enzyme activity associated with hepatocellular and cholestatic function. Module 6 (BUN and CREA) was associated with renal filtration and nitrogen metabolism. Correlations were generally stronger within modules than between modules, indicating that these physiological systems functioned relatively independently while contributing collectively to overall physiological variation.

Overall, these findings demonstrated that hematological and biochemical traits form integrated physiological networks rather than isolated variables. The identified modular framework provided a biological basis for subsequent PCA by defining higher-order physiological dimensions underlying individual variation in crossbred beef cattle.

### PCA

**Figure 3:**
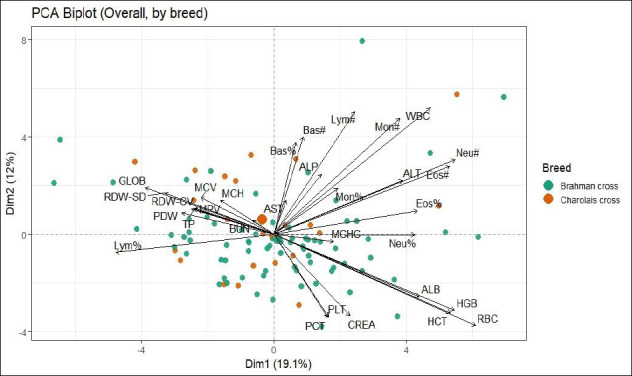
Principal component analysis biplot illustrating the multivariate structure of 31 hematological and biochemical traits in Brahman- and Charolais-crossbred cattle. Arrows represent variable loadings, whereas points represent individual animals colored according to breed. PC1 (19.1%) primarily reflected erythrocyte–renal variation, whereas PC2 (12.0%) represented immune–hepatic variation.

PCA of the 31 hematological and biochemical variables revealed a clear multivariate organization of physiological variation among cattle (Figure 3). The first two principal components explained 31.1% of the total variance, with PC1 accounting for 19.1% and PC2 accounting for 12.0%, indicating simultaneous contributions of multiple physiological systems to inter-individual variation.

PC1 contrasted erythrocyte-related parameters (RBC count, HGB, HCT, and MCHC) against renal-metabolic parameters (BUN and CREA), with positive and negative loadings, respectively. This component represented an erythrocyte–renal physiological gradient consistent with the modular organization identified in the correlation heatmap. PC2 was influenced predominantly by absolute leukocyte counts (WBC, Lym#, Neu#, Mon#, and Eos#) together with hepatic enzymes (ALT, AST, and ALP), representing an immune–hepatic activation axis. Additional contributions to PC2 were observed from protein metabolism variables (TP and GLOB) and erythrocyte volume-related indices (MCV, MCH, RDW-CV, and RDW-SD), representing nutritional and cellular-volume variation.

Breed distributions demonstrated substantial overlap, although slight separation along PC1 suggested subtle breed-associated differences in erythrocyte and renal-metabolic physiology. Variation along PC2 was associated more strongly with immune–hepatic status than with breed identity.

Overall, PCA confirmed that hematological and biochemical traits were structured into higher-order physiological dimensions aligned with the six physiological modules identified through correlation analysis. These findings highlighted coordinated interactions among hematological, hepatic, renal, and protein metabolism systems as major contributors to phenotypic variation in crossbred beef cattle. Because PCA revealed only limited breed separation, PERMANOVA was subsequently performed to evaluate subtle multivariate physiological differences between breeds.

## PERMANOVA

PERMANOVA based on Euclidean distances and 9,999 permutations was conducted using standardized hematological and biochemical variables to evaluate multivariate physiological differences between breeds (Table 3). A small but statistically significant breed effect was identified (F_1,95_ = 2.05, p = 0.028, R^2^ = 0.021). Breed explained only 2.1% of the total physiological variance, indicating that Brahman- and Charolais-crossbred cattle exhibited largely similar overall physiological profiles.

**Table 3:** Summary of permutational multivariate analysis of variance results.

Term	Degrees of freedom	Sum of squares	F	R²	p-value
Breed	1	62.92	2.052	0.02114	0.028*
Residual	95	2913.08	–	0.97886	–
Total	96	2976.00	–	1.00000	–

F = F statistic; R² = coefficient of determination

These findings suggested that environmental and management factors may exert greater influence than genetic background on blood profile architecture under tropical smallholder conditions. The PERMANOVA findings were consistent with PCA results demonstrating substantial overlap between breeds. Because overall multivariate separation was limited, additional analyses were conducted to identify individual variables contributing most strongly to breed-associated variation.

### Effect-size analysis of breed differences

**Figure 4:**
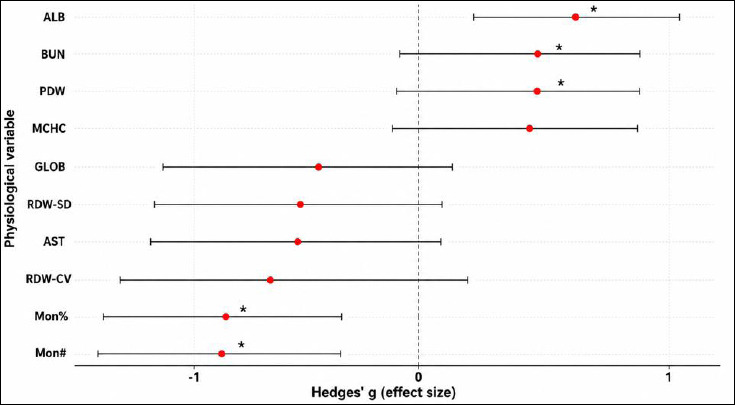
Forest plot showing the 10 hematological and biochemical variables with the largest breed-associated differences between Brahman- and Charolais-crossbred cattle based on Hedges’ g effect sizes and 95% confidence intervals. Asterisks indicate statistically significant differences between breeds (p < 0.05). ALB = albumin, AST = aspartate aminotransferase, BUN = blood urea nitrogen, GLOB = globulin, MCHC = mean corpuscular hemoglobin concentration, Mon# = monocyte count, Mon% = monocyte percentage, PDW = platelet distribution width, RDW-CV = red cell distribution width–coefficient of variation, RDW-SD = red cell distribution width–standard deviation.

Breed-associated differences among hematological and biochemical variables were further evaluated using standardized effect sizes (Hedges’ g). Figure 4 presents the 10 variables with the largest absolute effect sizes.

Monocyte-related indices exhibited the strongest breed-associated contrasts, with Mon% (g ≈ −0.81) and Mon# (g ≈ −0.82) significantly higher in Charolais-crossbred cattle than in Brahman-crossbred cattle. RDW-SD and RDW-CV demonstrated moderate negative effect sizes, indicating greater erythrocyte size variability in Charolais-crossbred cattle. In contrast, ALB, BUN, and PDW demonstrated positive effect sizes consistent with their relatively higher values in Brahman-crossbred cattle.

Most variables demonstrated small or negligible effect sizes (|g| < 0.20), supporting the overall finding of limited physiological divergence between breeds observed in PCA and PERMANOVA analyses.

Overall, descriptive, correlation-based, multivariate, and effect-size analyses consistently demonstrated that Brahman- and Charolais-crossbred cattle shared broadly similar hematological and biochemical profiles. The correlation network identified six conserved physiological modules, whereas PCA and PERMANOVA demonstrated only weak multivariate separation between breeds (R² < 0.03). Nevertheless, several biologically relevant differences were observed, particularly for monocyte-associated indices (Mon% and Mon#), erythrocyte distribution parameters (RDW-CV and RDW-SD), and selected hepatic and protein-nitrogen metabolites (ALT, ALP, BUN, and GLOB). Collectively, these findings suggested that although overall physiological architecture remained conserved between breeds, subtle differences persisted in immune regulation, erythrocyte morphology, and hepatic-metabolic function.

## DISCUSSION

This study introduces a modular systems-physiology framework for livestock blood profiling. By demonstrating that hematological and biochemical parameters in tropical crossbred beef cattle are organized into conserved and functionally coherent physiological modules rather than operating independently, the present findings extend beyond the traditional reductionist approach commonly applied in veterinary clinical pathology. The observation that breed explained <3% of the total multivariate variance further emphasizes the dominant influence of tropical smallholder environments on physiological organization and provides a new perspective for resilience-oriented livestock research. Previous studies involving Brahman or tropical cattle have largely focused on mean differences in individual parameters, whereas only limited studies have applied PCA or clustering approaches, and none have interpreted blood traits as integrated physiological modules under tropical smallholder production conditions.

### Physiological integration of blood traits under tropical conditions

Beef cattle reared in tropical environments are continuously exposed to multiple environmental stressors, including fluctuating nutrition, excessive heat, parasitic burdens, and endemic infectious diseases [29, 30]. Consequently, maintenance of physiological stability under these conditions is essential for survival and productive performance. Blood profiles provide clinically valuable and integrative indicators of physiological status because they reflect the combined activities of multiple biological systems, including erythropoiesis, immune surveillance, hepatic metabolism, and renal-nitrogen regulation. Therefore, understanding the interactions among these physiological systems and identifying differences between crossbred cattle types are important for detecting subclinical dysfunction, improving resilience-based selection, and supporting evidence-based herd management strategies [31, 32].

In the present study, a systems-physiology framework was applied to characterize multivariate hematological and biochemical phenotypes in Brahman- and Charolais-crossbred cattle raised under tropical smallholder conditions. The findings demonstrated that physiological variation was structured into coordinated modules rather than isolated traits, indicating that integrated systemic regulation underlies blood profile variation in tropical beef cattle.

### Physiological modules revealed by correlation structure

The correlation heatmap demonstrated that hematological and biochemical parameters clustered into six distinct physiological modules rather than being randomly distributed. These findings are consistent with previous reports showing that blood-based traits in cattle reflect coordinated physiological regulation [33, 34]. Similar multivariate structures have been observed in bovine studies where erythrocyte-related variables and leukocyte-associated indices formed independent clusters representing oxygen transport and immune regulation [33]. However, most previous investigations were conducted in dairy cattle maintained under temperate production systems, whereas the present study demonstrates comparable physiological modularity in tropical beef cattle raised under heterogeneous smallholder conditions.

The erythrocyte, platelet, and leukocyte modules indicated that hematopoietic functions were compartmentalized into biologically connected systems associated with oxygen transport, hemostasis, and immune activity. This observation aligns with clinical evidence indicating that these systems are highly coordinated, particularly under environmental stress conditions. Conversely, the protein metabolism, hepatic enzyme, and renal modules appeared relatively independent from hematological variables, suggesting greater physiological autonomy of metabolic and nitrogen-filtration pathways [17].

The modular organization observed in this study suggests that blood parameters should not be interpreted as isolated indicators but rather as interconnected physiological networks that collectively explain systemic variation. Importantly, the conserved modular structure identified in Brahman- and Charolais-crossbred cattle highlights the physiological plasticity of tropical beef cattle and suggests that common environmental pressures may exert stronger effects than breed-specific factors under tropical production conditions [27, 35].

### Multivariate physiological dimensions revealed by PCA

PCA further clarified how these physiological modules collectively contributed to variation among individual animals. PC1 primarily represented an erythrocyte–renal physiological axis opposing RBC-associated indices against renal-nitrogen variables. Clinically, this axis likely reflects coordinated regulation between oxygen-carrying capacity and renal-metabolic activity. Such variation is particularly relevant under tropical conditions, where hydration status, heat load, and metabolic demand fluctuate substantially. The alignment between PC1 and the erythrocyte and renal modules further supports previous reports indicating multisystem physiological coordination in cattle [17, 36, 37].

PC2 represented an immune–hepatic physiological axis driven predominantly by leukocyte-associated variables and hepatic enzyme activities. This pattern likely reflects coordinated physiological responses to inflammation, parasitic exposure, and metabolic stress, where immune activation and hepatic-metabolic processes change simultaneously under environmental pressure. The overlap between PC2 and the leukocyte and hepatic modules confirms that immune surveillance and hepatic metabolism function as interconnected physiological networks rather than isolated systems.

Substantial overlap between Brahman- and Charolais-crossbred cattle within the multivariate physiological space indicated that major sources of physiological variation were largely shared across crossbred groups [38]. These findings support the concept that environmental and management conditions exert stronger influences on baseline physiological organization than breed under tropical production systems [39, 40].

### Conserved physiological architecture across crossbred cattle

Although correlation analysis and PCA revealed clear physiological structuring, breed explained only a very small proportion of total physiological variance (R² < 0.03). This finding suggests that Brahman- and Charolais-crossbred cattle maintain a largely conserved baseline physiological architecture under tropical smallholder conditions [34, 41]. Biologically, this observation indicates that fundamental homeostatic processes, including hematopoiesis, immune regulation, metabolism, and renal function, remain strongly conserved across crossbred cattle exposed to similar environmental stressors [42].

The limited breed-associated effects identified in this study do not imply complete physiological uniformity between breeds. Rather, breed-associated variation appeared as subtle modifications within an otherwise conserved systemic physiological framework. Under chronic exposure to heat stress, nutritional variability, and endemic disease pressure, adaptive physiological mechanisms such as coordinated erythrocyte–renal balance and immune–hepatic regulation appear to be maintained across crossbred populations. This phenomenon may represent convergent physiological adaptation in which maintenance of systemic homeostasis becomes more important than breed-specific specialization under environmental stress [34].

From a clinical perspective, the conserved physiological architecture identified in this study suggests that shared physiological modules may provide a standardized framework for interpretation of blood profiles in tropical beef cattle herds. Although breed-specific physiological signals may still emerge under severe stress or disease conditions, the modular physiological approach offers potential value for herd-level health monitoring and detection of subclinical physiological imbalance across crossbred populations [7, 16, 43].

### Interpretation of univariate hematological and biochemical differences

Despite the conserved multivariate physiological architecture, several localized univariate differences were identified between breeds. Among hematological variables, Mon% and Mon# were significantly higher in Charolais-crossbred cattle [16, 28, 34]. These findings may reflect subtle differences in baseline immune activity or sensitivity to environmental and management-associated stressors rather than overt pathological conditions.

Among biochemical variables, only ALB and AST differed significantly between breeds, suggesting modest variation in protein metabolism and hepatic or muscular metabolic activity [44]. Increased ALB concentrations in Brahman-crossbred cattle may indicate differences in hepatic synthetic function or protein turnover, whereas elevated AST activity in Charolais-crossbred cattle likely reflects increased metabolic demand rather than hepatic damage because other hepatic and renal variables remained unchanged [45, 46].

Importantly, these univariate differences accounted for only a very small proportion of overall multivariate physiological variation (PERMANOVA R² = 0.021). Therefore, breed-associated variation appeared to occur within an otherwise conserved physiological regulatory framework. Collectively, these findings further support the conclusion that environmental and management conditions exert stronger effects on baseline physiology than breed in tropical beef cattle [47, 48].

### Clinical implications for herd health monitoring and management

Identification of a conserved modular physiological architecture provides a clinically relevant framework for herd health monitoring in tropical beef production systems. Rather than interpreting blood parameters individually, physiological assessment should consider integrated system-wide modules. This perspective aligns with established concepts in bovine clinical pathology, where blood profiles are considered highly sensitive indicators of systemic physiological disturbance and early subclinical dysfunction [7].

The modular approach may be especially useful in crossbred cattle populations that demonstrate enhanced adaptability and resilience under tropical conditions [49]. Simultaneous alterations within erythrocyte and renal modules may indicate disturbances in hydration status, oxygen delivery, or metabolic balance, whereas coordinated changes within leukocyte and hepatic modules may indicate inflammatory responses, parasitic burden, or metabolic stress [15, 50].

The conserved physiological architecture observed in Brahman- and Charolais-crossbred cattle may therefore represent a generalized adaptive mechanism to environmental stressors such as heat stress [22, 51, 52]. Consequently, routine blood profiling interpreted within a modular systems framework could support earlier detection of physiological imbalance and improve herd health management decisions in tropical smallholder production systems.

### Practical biomarkers and applications

Several blood variables identified in the present study may serve as practical biomarkers for field-level physiological monitoring in tropical beef cattle. Monocyte-associated indices (Mon% and Mon#) together with erythrocyte distribution parameters (RDW-CV and RDW-SD) may reflect differences in immune tone, erythropoietic regulation, and physiological stress in Charolais-crossbred cattle [53–55]. Similarly, ALB, BUN, and GLOB may provide useful information regarding protein-nitrogen balance and hepatic synthetic activity in Brahman-crossbred cattle [56].

Although these biomarkers function within broader physiological networks, they may provide useful complementary indicators for monitoring physiological status and identifying early subclinical imbalance in tropical beef herds [36, 38].

### Strengths and limitations

One of the major strengths of this study is the integration of descriptive, correlation-based, and multivariate analytical approaches to characterize coordinated physiological regulation in tropical beef cattle. This systems-physiology framework aligns with holistic concepts in bovine clinical pathology [7, 57]. In addition, this study addressed an important knowledge gap by demonstrating conserved modular physiological organization in crossbred beef cattle under tropical field conditions. The findings therefore provide clinically relevant information for herd health surveillance and physiological monitoring in tropical production systems.

Nevertheless, several limitations should be considered. First, the cross-sectional design evaluated physiological status at only a single time point and therefore could not account for temporal or seasonal physiological dynamics, disease exposure, or management-associated changes [34, 58]. Second, unequal sample sizes between crossbred groups may have reduced statistical sensitivity for detecting subtle breed-associated effects. Third, environmental and management conditions were not fully standardized among farms. Detailed data regarding diet composition, parasite burden, and direct indicators of heat stress were not available, thereby limiting assessment of specific environmental drivers underlying physiological variation.

Furthermore, standard hematological and biochemical parameters may not fully capture additional physiological signals associated with oxidative stress, acute-phase responses, endocrine regulation, or molecular biomarkers. Inclusion of additional metabolic variables such as glucose, electrolytes, non-esterified fatty acids, or β-hydroxybutyrate could further improve interpretation of physiological modules. Future studies incorporating acute-phase proteins, oxidative stress markers, and manual leukocyte differentials may provide deeper insights into immune and metabolic regulation in tropical beef cattle.

### Future research directions

Future longitudinal studies are needed to evaluate physiological dynamics and resilience throughout different production stages and environmental conditions. Integration of blood-based biomarkers with productivity, reproductive, and health-related data may facilitate identification of biomarkers associated with disease resistance, stress tolerance, and production efficiency.

Additionally, mechanistic investigations combining physiological profiling with genomic, transcriptomic, and metabolomic approaches together with controlled nutritional and environmental studies may help clarify the biological mechanisms underlying immune, hepatic, and protein-nitrogen variation in tropical beef cattle. Translation of modular physiological patterns into digital decision-support systems may further contribute to precision livestock farming and improve herd health management in tropical smallholder production systems.

## CONCLUSION

This study demonstrated that hematological and biochemical parameters in Brahman- and Charolais-crossbred beef cattle raised under tropical smallholder conditions are organized into conserved physiological modules rather than functioning as isolated variables. Correlation analysis identified six major physiological modules associated with erythrocyte regulation, platelet activity, leukocyte dynamics, protein metabolism, hepatic enzyme activity, and renal function. PCA further confirmed that physiological variation was structured into integrated higher-order dimensions dominated by erythrocyte–renal and immune–hepatic interactions. Although several univariate differences were observed, particularly for Mon%, Mon#, ALB, and AST, PERMANOVA demonstrated that breed contributed only minimally to total physiological variance (R² = 0.021), indicating that environmental and management conditions exert stronger influences than breed on physiological organization under tropical smallholder systems.

The findings of this study have important practical implications for herd health monitoring and veterinary management. The identified modular physiological framework supports the use of integrated blood profiling rather than isolated interpretation of individual hematological or biochemical parameters. Such an approach may improve early detection of subclinical physiological imbalance, enhance monitoring of stress adaptation and metabolic stability, and facilitate evidence-based herd management strategies in tropical beef production systems. The identified biomarkers, particularly monocyte-associated indices and erythrocyte distribution parameters, may also provide useful complementary indicators for field-level physiological assessment in crossbred cattle.

One of the major strengths of this study was the integration of univariate, correlation-based, and multivariate analytical approaches to characterize systemic physiological organization in tropical beef cattle under real-world field conditions. In addition, this study addressed a major knowledge gap by demonstrating conserved modular physiological architecture in crossbred cattle raised under tropical smallholder systems. However, several limitations should be acknowledged. The cross-sectional design evaluated animals at a single time point and therefore could not account for temporal physiological changes. Unequal sample sizes between breeds may also have reduced sensitivity for detecting subtle breed-associated effects. Furthermore, environmental variables such as diet composition, parasite burden, and direct indicators of heat stress were not comprehensively evaluated.

Future studies should incorporate longitudinal monitoring across production stages and seasons to better understand physiological adaptation and resilience over time. Integration of hematological and biochemical profiles with genomic, transcriptomic, metabolomic, and productivity-related data may further clarify the biological mechanisms underlying physiological regulation in tropical beef cattle. Additional incorporation of oxidative stress markers, acute-phase proteins, and endocrine indicators may also improve interpretation of systemic physiological responses under tropical production conditions.

Overall, this study provides the first evidence that hematological and biochemical traits in tropical crossbred beef cattle are organized into conserved physiological modules reflecting integrated system-level regulation. The results support the adoption of modular blood profiling as a practical and biologically meaningful approach for herd health monitoring, resilience assessment, and precision management in tropical smallholder beef production systems.

## DATA AVAILABILITY

The data supporting the findings of this study can be obtained from the corresponding author.

## AUTHORS’ CONTRIBUTIONS

ST: Conceptualization, methodology, data analysis, validation, integration, project administration, and writing – original draft. JT and PS: Data collection and methodology. PR: Conceptualization, methodology, and validation. PP and PI: Statistical analysis, data analysis, and validation. KS: Data collection. SS: Conceptualization, methodology, data analysis, validation, integration, supervision, and manuscript review and editing. All authors have read and approved the final version of the manuscript.
